# Industrial-Scale Preparation of Akebia Saponin D by a Two-Step Macroporous Resin Column Separation

**DOI:** 10.3390/molecules17077798

**Published:** 2012-06-26

**Authors:** Yue Wu, De Ji, Yunfei Liu, Chunfeng Zhang, Zhonglin Yang

**Affiliations:** State Key Laboratory of Natural Products and Functions, China Pharmaceutical University, Ministry of Education, No. 24 Tongjiaxiang, Nanjing 210009, China; E-Mails: ywupub@gmail.com (Y.W.); njjide@163.com (D.J.); liuyunfei05436@163.com (Y.L.)

**Keywords:** akebiasaponin D, *DipsaciRadix*, large-scale preparation, macroporous resin

## Abstract

A simple and efficient procedure for the industrial preparation of akebia saponin D, one of the bioactive compounds commonly found in the well-known Chinese Medicinal herb *DipsaciRadix,* was developed. First, HPD-722 was selected from among 10 kinds of macroporous absorption resins. Following this step, the purity of akebia saponin D was increased about 10 times from 6.27% to 59.41%. In order to achieve a higher purity, ADS-7 was chosen from among five kinds of macroporous absorption resins, and the purity of akebia saponin D was increased from 59.41% to 95.05%. The result indicated HPD-722 and ADS-7 were the most suitable resins to purify akebia saponin D from *DipsaciRadix*. Under these conditions, large-scale preparation of akebia saponin D was carried out successfully. The preparation method is simple, efficient, and has been demonstrated to be effective for large scale preparations of akebia saponin D from *DipsaciRadix*.

## 1. Introduction

*Dipsaci Radix*, Chinese name *XuDuan*, the dried root of *Dipsacusasper* Wall (Dipsacaceae) widespread in China, was first recorded in the ancient pharmaceutical book “Shen Nong Ben Tso Ching” and has been used since antiquity to treat bone diseases. It’s been reported that *DipsaciRadix* contains triterpenoid saponins [1,2,3] and iridoid glucosides [4,5]. Pharmacological studies have demonstrated that *DipsaciRadix* possesses various bio-active effects, including osteoprotective [6,7], inhibition of Alzheimer’s disease [8], anticomplementary [9], antinociceptive [10] and cytotoxic [11] activities.

**Figure 1:**
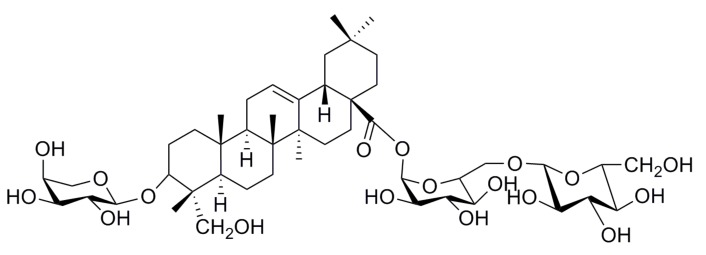
The chemical structure of ASD.

Akebia saponin D (3-*O*-α-L-arabinopyranosyl hederagenin-28-β-D-glucopyranoside-(1→6)-β-D-glucopyranoside, also called asperosaponin VI, ASD, Figure 1), the principal bioactive component in *Dipsaci Radix*, has been reported to have neuroprotective [12], osteoprotective [13], cardioprotective [14], and apoptosis-inducing effects [15].

The conventional methods to separate ASD from medicinal plants are column chromatography with silica gel [14,15,16,17] and preparative reversed phase HPLC [3]. However, these methods are not suitable for large-scale preparation because of the large amount of solvent wastage, poisonous residual solvents (chloroform and methanol, *etc*.), high cost, *etc*. Thus, an industrial-scale process for the separation of ASD is urgently needed.

In recent years, macroporous adsorption resins (MARs) have been gaining focus for the separation of herbal crude extracts in laboratorial and industrial applications, because they have the unique adsorption properties, including ideal pore structure, various surface functional groups, low operation expense, less solvent consumption, and easier regeneration [18,19,20,21,22]. In this paper, an efficient way to separate of ASD from *DipsaciRadix* using MARs was established. When the separation process was scaled up to the industrial-scale, the purity of ASD was greater than 90%.

## 2. Results and Discussion

### 2.1. Adsorption and Desorption Capacities, and Desorption Ratio Test on the MARs

In our preliminary study, it was impossible to separate ASD with only one kind of MAR. Based on the MAR specifications from the MAR producer, we designed a two-step separation, and 10 kinds of MARs were investigated in the first step, and five kinds of MARs in the second step, respectively.

**Figure 2:**
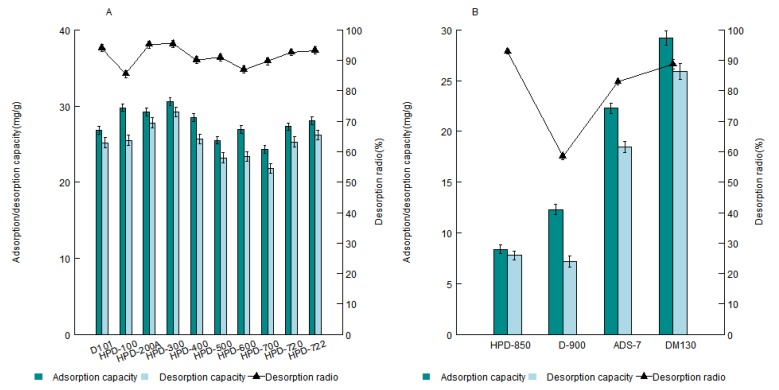
(**A**) Adsorption/desorption capacities and desorption ratio of ASD on the resins for the first step; (**B**) Adsorption/desorption capacities and desorption ratio of ASD on the resins for the second step.

In the static adsorption/desorption tests, the adsorption/desorption properties of resins for separating ASD in the first step were studied. As shown in Figure 2A, HPD-200A and HPD-300 had good adsorption/desorption performance. Next the adsorption/desorption properties of resins for purifying ASD in the second step were studied. DM130 had the best adsorption/desorption behavior. The result is shown in Figure 2B.

According to the static adsorption/desorption capacity results, HPD-200A and HPD-300 could be chosen for the first step, and DM130 for the second step, but the purity of ASD could not go to 90% using the combination of the selected resins, so the dynamic desorption experiment on all the testing resins was carried out, the resins were chosen according to the purities of ASD in the desorption solutions.

### 2.2. Purity Test in the Dynamic Desorption Experiment

In our preliminary research, ASD on resins could not eluted by less than 30% ethanol solution, but could be eluted by concentrations of ethanol over 50%, so in order to simplify the study, water, 30% and 60% ethanol solution were used as elution solvents, and the purity of ASD in fractions of 60% ethanol desorption solution was studied.

**Figure 3:**
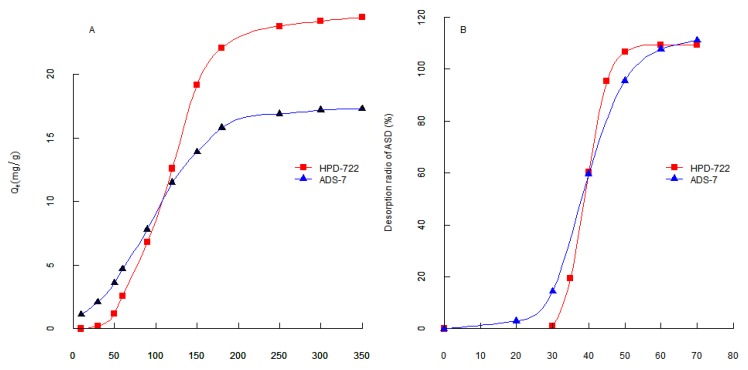
(**A**) Adsorption kinetics curves for ASD on HPD-722 and ADS-7 resins; (**B**) Dynamic desorption tests of ASD on HPD-722 and ADS-7 resins.

From the result of the purity test in the first step, which is shown in Table 1, the purity of ASD in the desorption solution of HPD-722 was the highest. Therefore, HPD-722 was selected as the adsorbing resin in the first step of separation. Judging from the result of the purity test in the second step (Table 1), ADS-7 was the best among the resins, so ADS-7 was chosen for the second step of separation, and the result is illustrated in Figure 3B.

**Table 1:** Purities of ASD in 60% ethanol desorption solutions in the dynamic desorption test.

Step	Resin	Purity (%, *x* ± SD, n = 3)
Step 1	D101	45.78 ± 4.36
	HPD-100	64.98 ± 3.69
	HPD-200A	65.87 ± 5.43
	HPD-300	66.81 ± 5.00
Step 1	HPD-400	52.17 ± 4.58
	HPD-500	50.46 ± 6.37
	HPD-600	50.69 ± 2.83
	HPD-700	48.05 ± 5.62
	HPD-720	47.54 ± 4.94
	HPD-722	70.08 ± 4.37
Step 2	HPD-850	72.43 ± 4.71
	D-900	72.10 ± 6.43
	ADS-7	89.40 ± 5.02
	DM130	78.82 ± 4.16
	HPD-722	77.25 ± 5.67

### 2.3. Adsorption kinetics on HPD-722 and ADS-7

In the present study, dynamic adsorption tests were conducted on the selected resins HPD-722 and ADS-7, the adsorption kinetic curves were obtained, adsorption equilibration time of ASD on each resin were obtained according to the adsorption kinetic curves. The adsorption capacities of ASD on HPD-722 and ADS-7 increased with the extension of adsorption time until equilibrium, as shown in Figure 3A. The adsorption capacity rapidly increased in the first 100 min, and then slowly increased. Finally, the adsorption capacity reached equilibrium after 200 min on HPD-722 resin, 180 min on ADS-7 resin.

### 2.4. Dynamic Desorption Curves on HPD-722 and ADS-7

In order to optimize the desorption conditions, the concentrations of ethanol solution to desorb ASD from HPD-722 and ADS-7 resins were investigated. After adsorption equilibrium was reached, the adsorbate-laden column was washed with water and different concentrations of ethanol (from 10% to 70%) and the dynamic desorption curves of ASD on HPD-722 and ADS-7 were obtained.

The desorption profiles of ASD on HPD-722 and ADS-7 are depicted in Figure 3B. The desorption ratio of ASD on resin increased with increasing ethanol concentration. On HPD-722, ASD couldn’t be desorbed from resin with 30% ethanol solution, but the desorption increased sharply when the ethanol concentration was over 40%, and reached a peak value at 50% ethanol. Hence, in the separation process on HPD-722, 30% ethanol solution could be applied to remove impurities and 50% ethanol solution could be used to desorb the ASD. The dynamic desorption test was also carried out on ADS-7 resin. 20% Ethanol solution could be applied to remove impurities from ASD, and 50% ethanol solution could be used to desorb the ASD. When the procedure was scaled up, the purity of ASD went down to below 90%. However, after replacing 20% ethanol solution with 30%, the purity went back to above 90%. Thus, 30% and 50% ethanol solution were finally selected as the desorption solutions on ADS-7 resin.

**Figure 4:**
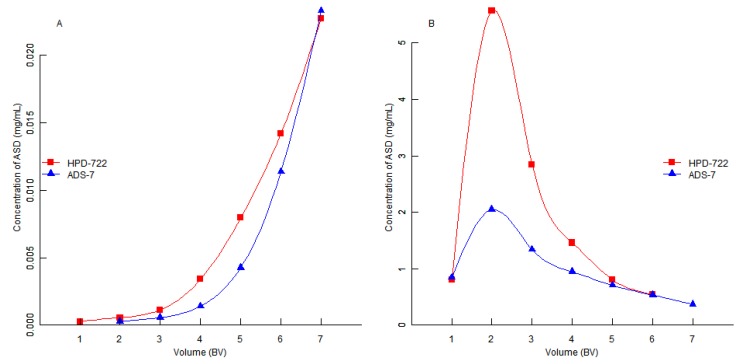
(**A**) The content of ASD *versus* volume of desorption solution on HPD-722 and ADS-7 resins at 30% ethanol solution; (**B**) The content of ASD *versus* volume of desorption solution on HPD-722 and ADS-7 resins at 50% ethanol solution.

In order to reduce the consumption of ethanol, the influence of volume of desorption solution was investigated. After adsorption equilibrium was reached, the resin was eluted with elution solutions at a flow rate of 2 bed volumes/h (BV/h), the content of ASD in the desorption solutions was plotted *versus* solution volume. As seen in Figure 4A,B, the HPD-722 resin could be washed with 6 BV 30% ethanol solution to remove highly polar impurities in the herb extract, then ASD was desorbed by 6 BV of 50% ethanol solution. In the second step, the ADS-7 resin could be washed with 6 BV 30% ethanol solution, then ASD was desorbed by 6 BV 50% ethanol solution.

In summary, a two-step procedure to separate ASD from *DipsaciRadix* with MARs was established in the laboratory. In the first step, a HPD-722 resin column was used, 6 BV water, 6 BV 30% ethanol solution and 6 BV 50% ethanol solution were used to wash the HPD-722 resin, the purity of ASD was increased from 6.27% to 72.03%. In the second step, 6 BV 30% ethanol solution and 6 BV 50% ethanol solution were used to wash the ADS-7 resin, and the purity of ASD was increased from 72.03% to 93.1%.

### 2.5. Industrial-Scale Preparation

**Figure 5:**
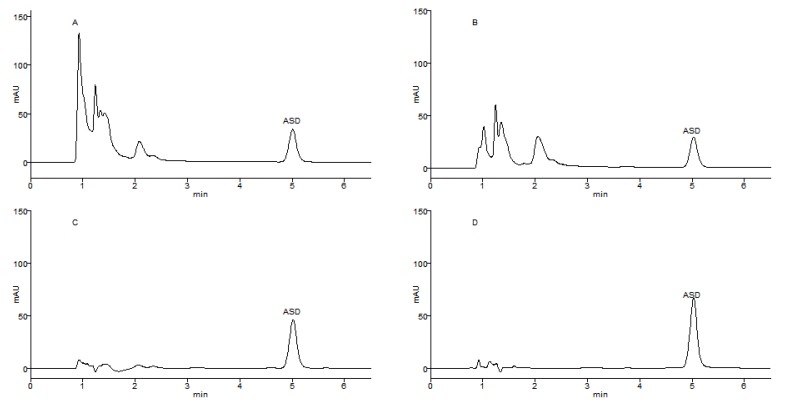
HPLC chromatograms of the loading solution of HPD-722 (**A**), the loading solution of ADS-7 (**B**), the purified ASD (**C**), and ASD standard (**D**).

In order to validate whether the method was applicable for industry, large-scale preparation of ASD from *DipsaciRadix* was carried out under the above-mentioned conditions. In the first step, a stainless steel column filled with HPD-722 resin (75 kg, 120 L BV) was used to separate ASD from the crude herb extract (concentration was 6.27 mg/mL, as calculated from the ASD content). Six BV distilled water, 6 BV 30% ethanol solution and 6 BV 50% ethanol solution were used to wash the column successively. The desorption solution of 50% ethanol solution was collected and concentrated under reduced pressure, the purify of ASD in the fraction was 59.41%, and a recovery of 98.07% was reached after this step. In the second step, 6 BV 30% ethanol solution and 6 BV 50% ethanol solution were used to wash the column packed with ADS-7 resin, 932 g light yellow powder containing 95.05% ASD was produced, a recovery of 57.62% was reached, so the total recovery in two steps was 56.51%. The large-scale preparation was successfully repeated four times. The HPLC chromatograms in each step are shown in Figure 5.

## 3. Experimental

### 3.1. Reagents and Materials

Acetonitrile was of HPLC grade (Merck, Darmstadt, Germany). Ethanol was of analytical grade and purchased from Dingxin Chemical Factory (Nanjing, China), distilled water was used. The standard sample of ASD was supplied by the Food and Drug Administration of Zhejiang Province (China). *DipsaciRadix* was purchased from a local Chinese medicine store in Sichuan Province (China) and identified by Zhonglin Yang (China Pharmaceutical University, Nanjing, China).

### 3.2. Absorbents

MARs tested including D101, HPD-100, HPD-200A, HPD-300, HPD-400, HPD-500, HPD-600, HPD-700, HPD-720, HPD-722, HPD-850, ADS-7, D-900, and DM130, purchased from Cang Zhou Bon Adsorber Technology Co., Ltd. (Hebei, China). The physical and chemical properties of the different resins are listed in Table 2. Before the experiments, the weighed resins were pretreated by soaking overnight in 95% ethanol, and thoroughly washed with distilled water to remove the monomers and porogenic agents trapped inside the pores during the synthesis process, in addition ADS-7, DM130, and D900 were pretreated successively with 1 mol/L HCl and NaOH solutions. The moisture content of each resin was calculated after the mass of resins dried at 110 °C was constant (Table 2).

**Table 2:** Physical and chemical properties of the MARs.

Trade name	Type of skeletons	Surface area (m^2^/g)	Average pore (nm)	Particle diameter (mm)	Polarity	Water content (%)
D101	Styrene copolymer	≥400	100-110	(0.3-1.25 mm)	Non-polar	65-75
HPD-100	Styrene copolymer	650-700	85-90	(0.315-1.25 mm) ≥90%	Non-polar	65-75
HPD-200A	Styrene copolymer	-	-	-	-	65-75
HPD-300	Styrene copolymer	800-870	50-55	(0.3-1.2 mm) ≥90%	Un-polarity	65-75
HPD-400	Styrene copolymer	500-550	75-80	(0.3-1.2 mm) ≥90%	Neutral-polarity	65-75
HPD-500	Styrene copolymer	500-550	55-75	(0.3-1.2 mm) ≥90%	Polarity	65-75
HPD-600	Styrene copolymer	550-600	80	(0.3-1.2 mm) ≥90%	Polarity	65-75
HPD-700	Styrene copolymer	650-700	85-90	(0.3-1.2mm) ≥90%	Un-polarity	65-75
HPD-720	Styrene copolymer	-	-	-	-	65-75
HPD-722	Styrene copolymer	485-530	130-140	(0.3-1.2 mm) ≥90%	Weak polarity	65-75
HPD-850	-	1,100-1,300	85-95	(0.3-1.2 mm) ≥90%	-	55-65
D-900	Methyl acrylate copolymer	-	-	(0.315-1.25 mm) ≥90%	Alkalescence	55-65
ADS-7	-	≥100	25-30	(0.3-1.25 mm)	High polarity	50-60
DM130	-	500-550	500-550	(0.3-1.25 mm)	Middle polar	65-75

### 3.3. Extraction of ASD and Sample Solutions Preparation

Dry powder of *DipsaciRadix* (25.0 kg) with a ASD content of 6.27% was extracted three times with 90% ethanol (ratio of solvent and crude herb = 10:1, v/w) under reflux for 2 h each time. The extract was then filtered, collected and evaporated under reduced pressure at 60 °C, the concentrated extract was obtained and photophobic stored in refrigerator at −4 °C. The ASD standard was dissolved in methanol to get a stock solution with concentration of 1 mg/mL, which was then diluted with mobile phase to seven different concentrations for the calibration plots and verification of the analysis method. The sample solution was prepared by diluting the stock soluton (concentration was 1 mg/mL, calculated by the ASD content) of sample with mobile phase.

### 3.4. Static Adsorption and Desorption Tests

The static adsorption and desorption tests were carried out to characterize the adsorption and desorption properties of the resins as follows: in the first step of separation, ten MARs: D101, HPD-100, HPD-200A, HPD-300, HPD-400, HPD-500, HPD-600, HPD-700, HPD-720, HPD-722 were selected for testing. The MARs (equal to 1.0 g dry weight) were placed into flasks with a lid, and 50 mL sample solutions (concentration was 6.27 mg/mL, calculated by the ASD content) were added, respectively. Subsequently, the flasks were continually shaken (160 rpm) for 6 h at 25 °C. The supernatant after adsorption was analyzed by HPLC. After adsorption equilibrium was reached, the solutions in the flasks were removed, and the resins were washed three times with distilled water. The desorption processes were then carried out. The resins were desorbed with 50 mL 70% ethanol solution. The flasks were shaken (160 rpm) for 6 h at 25 °C, then the desorption solutions were analyzed by HPLC. The adsorption capacities, desorption capacities and desorption ratios for ASD were calculated on each resin.

In the second step, five MARs, ADS-7, D-900, HPD-722, HPD-850, DM130, were tested. The resins (equal to 1.0 g dry resin) were placed into flasks with a lid, and 50 mL sample solutions (concentration was 6.27 mg/mL, calculated by the ASD content) were added, respectively. The static adsorption and desorption tests were then carried out as the above description. The desorption solutions were then collected to analyze.

The adsorption capacity, desorption capacity and desorption ratio of the resins for the resin were calculated according to the following Equations (1-3), respectively:

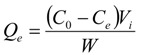
(1)

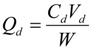
(2)

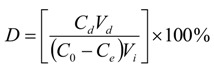
(3)
where Q_e_ is the adsorption capacity (mg/g dry resin) at adsorption equilibrium; C_0_ and C_e_ are the initial and equilibrium concentrations of ASD solutions, respectively (mg/mL), C_d_ represents the concentration of ASD in the desorption solution (mg/mL). V_i_ and V_d_ are the volume of the initial sample and desorption solution (mL), respectively. W is the weight of the dry resin used (g); Q_d_ is the desorption capacity; D is the desorption ratio (%).

### 3.5. Dynamic Adsorption Experiments

To evaluate the equilibration time of ASD in contact with resins, dynamic adsorption experiments were carried out in a glass column (2 cm × 45 cm) wet packed with 20 g (dry weight) resin with 40 mL of the BV. Sample solutions were loaded onto the glass column at 1 BV/h, the adsorption equilibriums were reached when the concentration of ASD monitored by HPLC became constant, then the adsorption kinetic curves were obtained.

### 3.6. Dynamic Desorption Tests

Dynamic desorption experiments were carried out in a glass column (2 cm × 45 cm) wet packed with 20 g (dry weight) resin with 40 mL of the BV. In the first step, the extracted solution of crude herb (concentration was 0.1 g/mL) was used as the sample solution. In the second step, the concentrated fraction (6 mg/mL, calcuated by the ASD content) of 60% desorption solution from HPD-722 was used. After the sample solution was loaded onto the glass column at 1 BV/h, and the adsorption equilibrium was reached, the adsorbate-laden column containing resin was washed successively with distilled water and different concentrations of ethanol solution. The volume of each fraction was 6 BV, and the desorption solutions were directly analyzed by HPLC.

### 3.7. HPLC Analysis

The quantification of ASD was carried out on an Agilent 1260 series HPLC system (Agilent, Boblingen, Germany) equipped with an online vacuum degasser, a binary gradient pump and an autosampler. All the samples were filtered through a 0.45 µm syringe filter prior to injection into the HPLC system. Analysis was performed on a Agilent reversed-phase C18 column (100 mm × 4.6 mm, 3.5 µm) (Agilent, Palo Alto, CA, USA) at temperature 30 °C. The mobile phase was a mixture of acetonitrile and water (30:70), the flow-rate was 1 mL/min, the injection volume was 20 µL, the wavelength of DAD detector was 212 nm. A calibration curve of ASD was constructed using a series of concentrations of the standard solutions, and the regression equation for concentration and corresponding peak area was calculated in the form of Y = aX + b. The results indicated that the working calibration curve based on ASD standard solutions showed excellent linearity in the range of 57.6-500 µg/mL of akebia saponin D: (Y = 3.78X + 19.3, R^2^ = 0.9999). The inter-day and intra-day precisions for ASD expressed as relative standard deviation (RSD, n = 5) were lower than 2.0% and 5.0%, respectively. The quantitation limit of ASD was 1.0 µg/mL, and the mean recovery of ASD was 101.27 ± 1.04% (n = 6).

### 3.8. Large-Scale Preparation of ASD

The concentrated extract (concentration was 62.7 mg/mL, calculated from the ASD content, 25 L) from *DipsaciRadix* was diluted in water to afford a diluted extract solution (concentration was 6.27 mg/mL, calculated by the ASD content, 250 L), after 1 mL of the solution was sampled, diluted with mobile phase to 50 mL, and analyzed by HPLC, the solution was subjected to a stainless steel column (30.0 cm × 400.0 cm, containing 75.0 kg HPD-722) with 120 L of BV. The separation process was then taken on after adsorption for 1 h. Distilled water was used to wash the elution solution until the desorption solution was clear, then 6 BV 30% ethanol solution was used to remove the dark color and high polar components, the ASD was desorbed by 6 BV 50% ethanol, the flow rate of each gradient was 2 BV/h, the fraction of 50% ethanol desorption solution was collected and concentrated (concentration was 6 mg/mL, calculated by the ASD content) as the loading solution in the second step, 1 mL of the solution was sampled, diluted with mobile phase to 50 mL, and analyzed by HPLC. The second step was carried out on a stainless steel column (30.0 cm × 400.0 cm, containing 105.0 kg ADS-7) with 150 L of BV. The resin was eluted with 6 BV 30% ethanol solution and 6 BV 50% ethanol solution at a flow rate of 2 BV/h, successively. Then the fraction of 50% ethanol desorption solution was collected, concentrated, and dried, the purity of ASD was determined.

## 4. Conclusions

In the present study, a two-step method to separate ASD from *DipsaciRadix* was successfully established. According to the results of dynamic adsorption/desorption studies, HPD-722 and ADS-7 were considered to perform the best out of 14 kinds of MARs tested. In the first step, HPD-722 was used and washed with 6 BV water, 6 BV 30% ethanol, and 6 BV 50% ethanol, successively; the purity of ASD in this step was increased 10 times from 6.27% to 59.41%. Then ADS-7 was used and eluted with 6 BV 30% ethanol and 6 BV 50% ethanol successively, and the purity of the ASD was increased from 59.41% to 95.05%.
